# Cross-sectional comparison of cannabis use in adults with neuropathic versus non-neuropathic pain

**DOI:** 10.3389/fpain.2025.1677391

**Published:** 2025-12-18

**Authors:** Carl Joshua P. Laroya, Crystal Lederhos Smith, Ross J. Bindler, Michael G. McDonell, Jamie Lewis, Marian Wilson

**Affiliations:** 1 Northwest Spine and Pain Medicine, Spokane, WA, United States; 2 Elson S. Floyd College of Medicine, Washington State University, Spokane, WA, United States; 3 College of Nursing, Washington State University, Spokane, WA, United States; 4 Northwest Center for Regenerative Medicine, Northwest Spine and Pain Medicine, Spokane, WA, United States

**Keywords:** neuropathic pain, chronic pain, cannabis, tetrahydrocannabinol, cannabidiol, pain management, cannabis use patterns, PROMIS

## Abstract

**Introduction:**

Cannabis has been decriminalized by many states and shows promise in treating both neuropathic and non-neuropathic pain through its interaction with the endocannabinoid system and anti-inflammatory effects. This study examines differences in cannabis use for adults whose most bothersome chronic pain condition is neuropathic vs. non-neuropathic.

**Materials and methods:**

Survey data were collected from adults receiving care at a pain clinic. Participants completed demographic questions and standardized self-report measures (PROMIS Pain Intensity/Interference and the ID-Pain tool). Participants' most bothersome pain condition(s) were categorized as neuropathic or non-neuropathic pain based on ID-Pain scores. Linear regression models assessed differences in frequency and duration of cannabis product use between groups, adjusting for age and sex.

**Results:**

A total of 113 individuals were recruited; following exclusions and missing data, 104 participants (61.5% female) were included in the final analysis. Of these, 36.5% reported neuropathic pain as their most bothersome, and 63.5% reported non-neuropathic pain. Those with neuropathic pain reported significantly more days per month of Tetrahydrocannabinol/Cannabidiol (THC/CBD) combination (*b* = 5.96, *p* = 0.02), Cannabidiol-only (CBD-only) (*b* = 8.82, *p* = 0.03), and Tetrahydrocannabinol-only (THC-only) products (*b* = 7.04, *p* = 0.02). They also used THC-only (*b* = 0.97, *p* < 0.05) and THC/CBD (*b* = 1.09, *p* < 0.01) products more frequently per day. Neuropathic pain was positively associated with pain intensity (*b* = 4.10, *p* < 0.001) and interference (*b* = 4.95, *p* < 0.001).

**Discussion:**

Adults whose most bothersome pain condition(s) were neuropathic used cannabis, especially THC and THC/CBD combination products, more frequently than those whose most bothersome pain was non-neuropathic. Participants with neuropathic pain also reported higher levels of pain intensity and interference. Further longitudinal research is needed to confirm whether increased use of THC-rich cannabis provides symptom relief for adults with neuropathic pain.

## Introduction

Chronic pain impacts an estimated 20% of the global population, with over 100 million people affected in the United States (U.S.) (1, 2). This condition imposes a substantial burden on individuals and healthcare systems alike, necessitating effective and sustainable treatment strategies. Chronic pain can be broadly classified into neuropathic pain, caused by damage to the somatosensory nervous system, and non-neuropathic pain, stemming from nociceptive signaling due to response to actual tissue damage or potentially harmful stimuli (3, 4). Some conditions, such as cancer-related pain, may involve mixed mechanisms. The distinct pathophysiological mechanisms underlying these pain types suggest the need for differentiated therapeutic approaches.

Most states allow for legal medical or non-medical use of cannabis and some evidence supports its effectiveness in managing both neuropathic and non-neuropathic pain (5–7). Cannabis interacts with the endocannabinoid system, making it a potential treatment for neuropathic pain. In a systematic review, cannabis-based treatments, particularly nabiximols, are likely effective in reducing neuropathic pain in multiple sclerosis, although results vary by product and patient population (8). Similarly, a multicriteria decision analysis study found that cannabis-based medicinal products, especially those with a 1:1 tetrahydrocannabinol/cannabidiol (THC/CBD) ratio, achieved the highest scores in managing chronic neuropathic pain due to their positive impact on quality of life and favorable side effect profile (9). Neuropathic pain was managed more effectively with cannabis than with traditional pharmacotherapies like duloxetine and gabapentin (9). However, further trials are needed to confirm long-term safety and efficacy. For non-neuropathic pain, a range of studies suggest cannabis may offer benefit due to its anti-inflammatory and analgesic properties (10). For example, research has demonstrated improvement in hand function, grip strength, and reduced pain scores in patients with hand osteoarthritis (11), as well as reduced pain scores in cancer patients (12). Despite these findings demonstrating some benefits from cannabis use, significant gaps remain regarding the specific types of cannabis products that may improve health. Further research is needed to optimize cannabis use and address potential adverse effects and inconsistencies in effectiveness compared to non-use (13–15).

This study seeks to evaluate and compare cannabis use patterns among adults whose most bothersome pain condition(s) are neuropathic vs. non-neuropathic in nature, focusing on the differential use of THC- and CBD-only products. As part of a larger longitudinal observational study investigating health outcomes over time in U.S. adults experiencing pain, we analyzed baseline data to identify variations in cannabis use patterns and health outcomes within specific patient populations. In addition to examining pain type, we also aimed to explore whether pain symptom burden, specifically pain intensity and interference, helps explain cannabis use patterns. Prior research suggests that individuals with more severe or interfering pain are more likely to engage in frequent or intensive cannabis use (16, 17).

## Methods

People receiving care at a partnering interventional pain clinic in Washington State, U.S. were recruited into a longitudinal observational survey-based study. Eligible individuals provided informed consent hosted within the primary research university's online Research Electronic Data Capture (REDCap) (18) system. Key eligibility criteria included being an adult (21 years or older, or 18–20 years with a cannabis use authorization) experiencing pain and using any cannabis-based products without any significant medical conditions that would prohibit survey completion. Following verification by the research and clinic teams, participants were sent a baseline survey which included demographic information and cannabis/cannabis-product use information as well as valid and reliable self-report measures related to pain. Outcome measures included the ID-Pain tool (Pfizer) (19) to assess the presence of neuropathic pain as the most bothersome pain type, and Patient Reported Outcomes Measurement Informational System (PROMIS) Pain Intensity (20) and Interference (21). A cutoff score of ≥3 on the ID-Pain signifies neuropathic pain based on the tool's robust diagnostic performance, with an area under the ROC curve (AUC) of 0.89, indicating excellent diagnostic accuracy (19). Conditions like cancer-related pain were classified based on the predominant pain mechanism reported by the participant. Participants reporting only headache-related pain were excluded due to ambiguity in classification. Importantly, some participants may experience both neuropathic and non-neuropathic pain simultaneously; however, participants were asked to identify their most bothersome pain, and classification was based on this response.

After the first six months of recruiting, a cross-sectional data export of the baseline survey was created. Categorical variables were summarized with counts and frequencies while means and standard deviations were used for continuous variables. Linear regression models were used to evaluate the association between the presence of neuropathic pain and cannabis use; examining number of days per month, as well as number of times per day for each type of cannabis use. Models included covariates of age and birth sex. This approach was chosen over Analysis of Covariance (ANCOVA), although asymptotically equivalent, to allow for direct estimation and interpretation of regression coefficients for both categorical and continuous predictors. Also, linear regression allowed for the facilitation of modeling potential interaction effects, which we anticipated could be needed as we conducted a more detailed examination of the data. The study was reviewed and approved by the primary research university's institution review board (IRB). Good research and clinical principles were followed throughout the study.

We also conducted secondary models to assess how pain symptom burden, measured via PROMIS pain intensity and interference scores, varied by pain type. These models examined the association between neuropathic pain classification and PROMIS scores, controlling for age (22, 23) and sex (24, 25). This analytic approach helps clarify whether greater cannabis use among individuals with neuropathic pain may be related to higher levels of pain intensity and interference.

## Results

**Figure 1:**
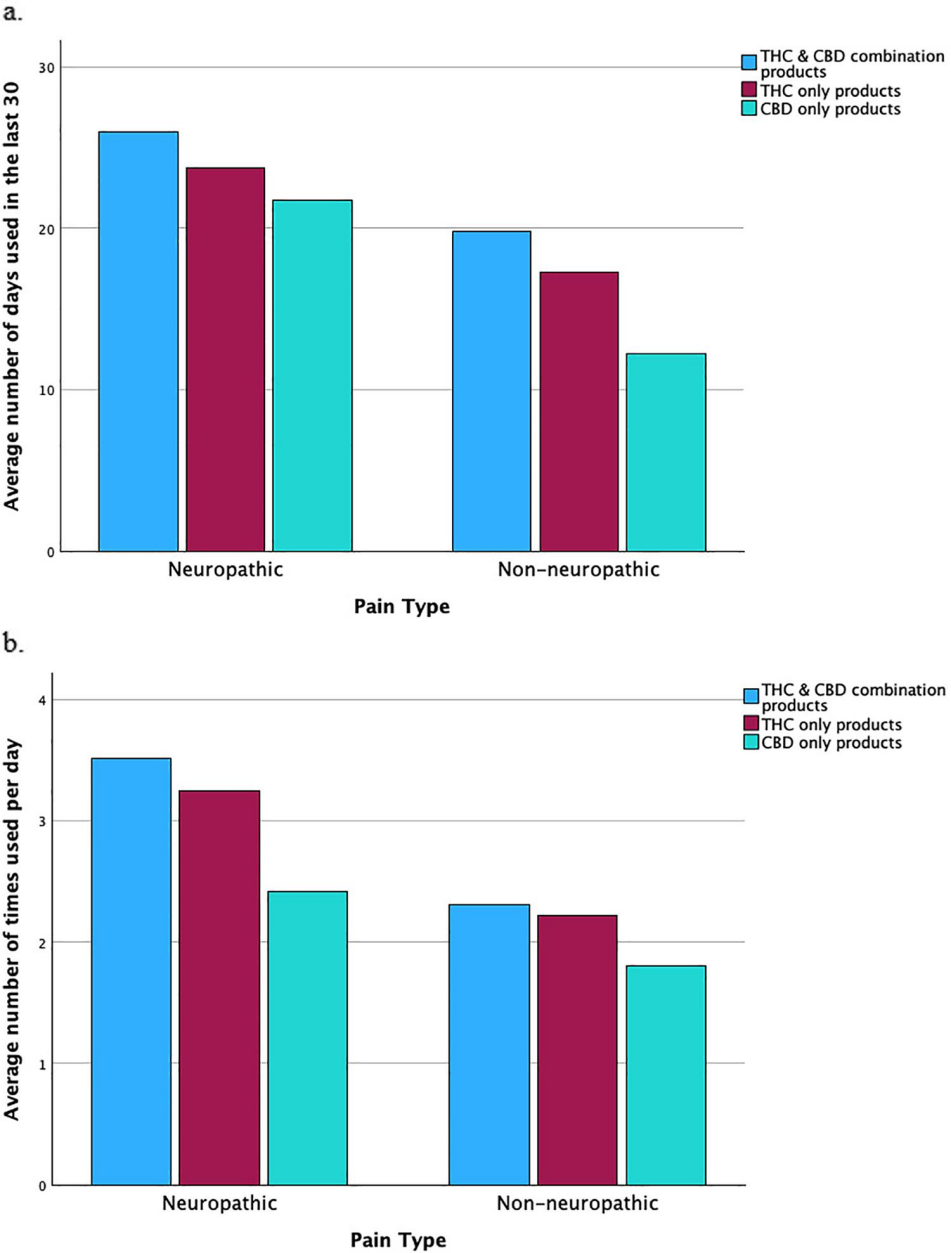
Cannabis use patterns among adults with neuropathic and non-neuropathic pain. **(a)** Average number of days per 30-day period that THC-only, CBD-only, and combination THC/CBD products were used by participants with neuropathic vs. non-neuropathic pain. **(b)** Average frequency of THC-only, CBD-only, and combination THC/CBD product use per day among participants with neuropathic vs. non-neuropathic pain.

A total of 113 individuals were recruited during the first six months of the study. Three participants reported experiencing only headache-related pain, which is not classified as neuropathic or non-neuropathic pain, leaving 110 participants for the final analysis; *n* = 42 (38%) with neuropathic pain and *n* = 68 (62%) with non-neuropathic pain. Most participants were female (61.5%), White (92%), and non-Hispanic/Latino/a/x, with an average age of 55 years (SD: 13.3). Participant characteristics, including PROMIS pain intensity and interference scores by pain type, are presented in Table 1. Overall, *n* = 91 (88%) of participants used products containing THC, whether in combination with CBD or without CBD, and *n* = 12 (12%) exclusively used CBD-only products (i.e., did not use THC at all; Table 2). See Figure 1a for a visual depiction of average number of days of cannabis use reported in the previous 30 days, by pain type and cannabis type. See Figure 1b for a visual depiction of average number of times per day of cannabis use reported, by pain type and cannabis type. Additionally, six individuals opted not to provide cannabis use information, resulting in data available to analyze for 104 participants referenced in Table 2.

**Table 1:** Participant Demographics.

Variable	Neuropathic pain (*n* = 42)	Non-neuropathic pain (*n* = 68)	Total sample (*n* = 113)
Age*	53 (12.6)	56 (13.8)	55 (13.3)
Gender			
Male	17 (44.7%)	22 (33.3%)	39 (37.5%)
Female	19 (50.0%)	41 (62.0%)	60 (57.7%)
Transgender	1 (2.6%)	0 (0.0%)	1 (1.0%)
Non-binary	1 (2.6%)	3 (4.5%)	4 (3.8%)
Birth sex			
Male	18 (47.4%)	22 (33.3%)	40 (38.5%)
Female	20 (52.6%)	44 (66.7%)	64 (61.5%)
Race			
Indigenous	1 (2.6%)	1 (1.5%)	2 (1.9%)
Asian American	0 (0.0%)	2 (3.0%)	2 (1.9%)
Black or African American	1 (2.6%)	3 (4.5%)	4 (3.8%)
White	34 (89.5%)	62 (93.9%)	96 (92.3%)
Other	2 (5.3%)	0 (0.0%)	2 (1.9%)
Ethnicity			
Hispanic and/or Latino/Latina/Latinx	0 (0.0%)	3 (4.5%)	3 (2.9%)
Non-Hispanic and/or Non-Latino/Latina/Latinx	33 (86.8%)	63 (95.5%)	96 (92.3%)
Marital status			
Married	13 (34.0%)	30 (45.5%)	43 (41.3%)
Remarried	1 (2.6%)	2 (3.0%)	3 (2.9%)
Widowed	1 (2.6%)	7 (10.6%)	8 (7.7%)
Separated	3 (7.9%)	2 (3.0%)	5 (4.8%)
Divorced	11 (28.9%)	16 (24.2%)	27 (26.0%)
Never Married	5 (13.2%)	8 (12.1%)	13 (12.5%)
Domestic Partnership	3 (7.9%)	0 (0.0%)	3 (2.9%)
Other (both self-declared “single”)	1 (2.6%)	1 (1.5%)	2 (1.9%)
Employment status			
Full Time	9 (23.7%)	12 (18.2%)	21 (20.2%)
Part Time	1 (2.6%)	7 (10.6%)	8 (7.7%)
Disabled	16 (42.1%)	23 (34.8%)	39 (37.5%)
Retired	8 (21.1%)	18 (27.3%)	26 (25.0%)
Student	1 (2.6%)	0 (0.0%)	1 (1.0%)
Homemaker/stay-at-home parent/caregiver	0 (0.0%)	4 (6.1%)	4 (3.8%)
Other	3 (7.9%)	2 (3.0%)	5 (4.8%)
Education level			
Less than High School	1 (2.6%)	2 (3.0%)	3 (2.9%)
Highschool diploma or GED	5 (13.2%)	9 (13.6%)	14 (13.5%)
Some College	16 (42.1%)	16 (24.2%)	32 (30.8%)
Associate Degree or Technical Certification	9 (23.7%)	17 (25.8%)	26 (25.0%)
Bachelor's Degree	6 (15.8%)	14 (21.2%)	20 (19.2%)
Graduate Degree	1 (2.6%)	5 (7.6%)	6 (5.8%)
Other	0 (0.0%)	3 (4.5%)	3 (2.9%)
Have you ever been diagnosed with a mental health disorder?			
Yes	22 (57.9%)	28 (42.4%)	50 (48.1%)
No	16 (42.1%)	37 (56.1%)	53 (51.0%)
Mental health diagnosis			
Bipolar I or II	5 (13.2%)	6 (9.1%)	11 (10.6%)
Major Depressive Disorder	9 (23.7%)	14 (21.2%)	23 (22.1%)
Generalized Anxiety Disorder	13 (34.2%)	13 (19.7%)	26 (25.0%)
Schizophrenia	1 (2.6%)	0 (0.0%)	1 (1.0%)
Other	10 (26.3%)	12 (18.2%)	22 (21.2%)
Pain conditions			
Arthritis	27 (71.1%)	35 (53.0%)	62 (59.6%)
Back Pain	33 (86.8%)	58 (87.9%)	91 (87.5%)
Cancer Related	3 (7.9%)	1 (1.5%)	4 (3.8%)
Complex Regional Pain Syndrome/Reflex Sympathetic Dystrophy	1 (2.6%)	0 (0.0%)	1 (1.0%)
Fibromyalgia	7 (18.4%)	14 (21.2%)	21 (20.2%)
Joint Pain	29 (76.3%)	36 (54.5%)	65 (62.5%)
Migraines/Head	15 (39.5%)	23 (34.8%)	38 (36.5%)
Neck Pain	29 (76.3%)	25 (37.9%)	54 (51.9%)
Nerve Pain/Neuropathy	28 (73.7%)	27 (40.9%)	55 (52.9%)
Post-Surgical Pain	11 (28.9%)	4 (6.1%)	15 (14.4%)
Other	7 (18.4%)	14 (21.2%)	21 (20.2%)
Pain Intensity *t*-score*	70.37 (5.56)	65.79 (5.32)	67.46 (5.82)
Pain Interference *t*-score*	70.08 (5.56)	65.49 (6.20)	67.13 (6.35)

Demographics are presented in *N* (%) unless marked with asterisk (*), wherein they are presented as mean (standard deviation).

**Table 2:** Baseline cannabis use characteristics.

Survey question	Neuropathic pain (*n* = 38)	Non-neuropathic pain (*n* = 66)	Total sample (*n* = 104)
Do you use cannabis product(s) that is/are a mix of THC/CBD such as whole leaf marijuana (known as a “bud”)?			
No	7 (18.4%)	20 (30.3%)	27 (26.0%)
Yes	31 (81.6%)	46 (69.7%)	77 (74.0%)
What type of THC/CBD product(s) do you use (SELECT ALL THAT APPLY)? [percentage reflects endorsement]			
Whole flower (also known as “bud”)	22 (57.9%)	32 (48.5%)	54 (51.9%)
Concentrate (product with excess plant materials removed)	18 (47.4%)	15 (22.7%)	33 (31.7%)
Edibles (cannabis products meant to be eaten or drunk)	27 (71.1%)	33 (50.0%)	60 (57.7%)
Tinctures (herbal solutions in an alcohol mixture)	6 (15.8%)	8 (12.1%)	14 (13.5%)
Topicals (cannabis infused product applied to skin)	16 (42.1%)	22 (33.3%)	38 (36.5%)
Other	0 (0.0%)	4 (6.1%)	4 (3.8%)
How do you administer the THC/CBD product(s) (SELECT ALL THAT APPLY)?			
Combustion	22 (57.9%)	31 (47.0%)	53 (51.0%)
Vaporizing	18 (47.4%)	19 (28.8%)	37 (35.6%)
Edible consumption	26 (68.4%)	31 (47.0%)	57 (54.8%)
Topical application	16 (42.1%)	24 (36.4%)	40 (38.5%)
Do you use cannabis product(s) that is/are a THC only?			
No	18 (47.4%)	33 (50.0%)	51 (49.0%)
Yes	20 (52.6%)	33 (50.0%)	53 (51.0%)
What type of THC only product(s) do you use (SELECT ALL THAT APPLY)? [percentage reflects endorsement]			
Concentrate (product with excess plant materials removed)	12 (31.6%)	14 (21.2%)	26 (25.0%)
Edibles (cannabis products meant to be eaten or drunk)	17 (44.7%)	20 (30.3%)	37 (35.6%)
Tinctures (herbal solutions in an alcohol mixture)	3 (7.9%)	2 (3.0%)	5 (4.8%)
Topicals (cannabis infused product applied to skin)	6 (15.8%)	9 (13.6%)	15 (14.4%)
Other	2 (5.3%)	6 (9.1%)	8 (7.7%)
How do you administer the THC product(s) (SELECT ALL THAT APPLY)?			
Combustion	15 (39.5%)	19 (28.8%)	34 (32.7%)
Vaporizing	11 (28.9%)	13 (19.7%)	24 (23.1%)
Edible consumption	17 (44.7%)	19 (28.8%)	36 (34.6%)
Topical application	6 (15.8%)	8 (12.1%)	14 (13.5%)
Other	1 (2.6%)	0 (0.0%)	1 (1.0%)
Do you use cannabis product(s) that is/are a CBD only?			
No	25 (65.8%)	40 (60.6%)	65 (62.5%)
Yes	12 (31.6%)	26 (39.4)	38 (36.5%)
What type of CBD only product(s) do you use (SELECT ALL THAT APPLY)? [percentage reflects endorsement]			
Concentrate (product with excess plant materials removed)	3 (7.9%)	6 (9.1%)	9 (8.7%)
Edibles (cannabis products meant to be eaten or drunk)	10 (26.3%)	15 (22.7%)	25 (24.0%)
Tinctures (herbal solutions in an alcohol mixture)	3 (7.9%)	5 (7.6%)	8 (7.7%)
Topicals (cannabis infused product applied to skin)	6 (15.8%)	17 (25.8%)	23 (22.1%)
Other	0 (0.0%)	1 (1.5%)	1 (1.0%)
How do you administer the CBD only product(s) (SELECT ALL THAT APPLY)?			
Combustion	5 (13.2%)	4 (6.1%)	9 (8.7%)
Vaporizing	3 (7.9%)	4 (6.1%)	7 (6.7%)
Edible consumption	10 (26.3%)	18 (27.3%)	28 (26.9%)
Topical application	6 (15.8%)	20 (30.3%)	26 (25.0%)

Demographics are presented in *N* (%) unless marked with asterisk (*), wherein they are presented as mean (standard deviation). Participants with “headache pain” did not fall into either neuropathic or non-neuropathic pain categories; thus, the number of participants in the total column is not the sum of those in the neuropathic and non-neuropathic columns. Due to allowing for multiple selections, rounding, and/or participant nonresponses, summing percentages across all categories may not equal exactly 100%.

### Number of days per month of cannabis use

The overall model examining the outcome of number of days per month of THC-only product use was significant, F (3, 48) = 3.48, *p* = 0.02, accounting for 18% of the variance (R^2^ = 0.18, adjusted R^2^ = 0.13). Participants with neuropathic pain used THC-only products seven more days per month [*b* = 7.04, 95% CI (1.02, 13.06), *p* = 0.02]. This model was the only model examined wherein age had a significant effect [*b* = −0.26, 95% CI (−0.50, −0.01), *p* = 0.04], indicating that for each 3.85 years older a participant was, they reported using THC approximately 1 less day per month.

Neither the overall model examining the outcome of number of days per month of combination THC/CBD products, F (3, 72) = 2.44, *p* = 0.07, nor the overall model examining the outcome of number of days per month of CBD only use, F (3, 33) = 2.38, *p* = 0.09, were significant; with these models accounting for 9% (R^2^ = 0.09, adjusted R^2^ = 0.05) and 18% (R^2^ = 0.18, adjusted R^2^ = 0.10) of the variance, respectively. The individual variable of neuropathic pain, however, was significant in both models, indicating that participants with neuropathic pain used combination THC/CBD products nearly six days more per month [*b* = 5.96, 95% CI (1.12, 10.80), *p* = 0.02], and used CBD-only products nearly nine days more per month [*b* = 8.82, 95% CI (1.10, 16.54), *p* = 0.03], compared to those with non-neuropathic pain. No covariates were statistically significant in these models.

### Number of times per day of cannabis use

The overall regression model examining the outcome of number of times per day of THC/CBD combination cannabis use was significant, F (3, 71) = 4.72, *p* < 0.01, accounting for 17% of the variance (R^2^ = 0.17, adjusted R^2^ = 0.13). In this model, neuropathic pain was significantly related to more frequent use per day [*b* = 1.09, 95% CI (0.39, 1.79), *p* < 0.01], such that people with neuropathic pain used THC/CBD combination products approximately one more time per day, compared to people with non-neuropathic pain. No covariates were statistically significant.

Overall regression models for number of times per day of cannabis use were not significant for either THC-only, F (3, 47) = 2.67, *p* = 0.06 (R^2^ = 0.15, adjusted R^2^ = 0.09) or CBD-only use, F (3, 33) = 1.97, *p* = 0.14 (R^2^ = 0.15, adjusted R^2^ = 0.08), respectively. The presence of neuropathic pain was, however, associated with a higher number of times per day of cannabis use in the THC-only model [*b* = 0.97, 95% CI (0.09, 1.85), *p* < 0.05], wherein participants with neuropathic pain used THC-only products nearly one more time per day than participants with non-neuropathic pain. There was no significant relationship between the neuropathic pain type and CBD-only product use per day [*b* = 0.64, 95% CI (−0.17, 1.46), *p* = 0.12] and no covariates were statistically significant.

### Pain intensity and interference

Our second aim examined relationships between neuropathic pain and PROMIS pain intensity and interference scores, controlling for age and birth sex. The overall pain intensity model was statistically significant, F (3, 104) = 7.00, *p* < 0.001, and accounted for approximately 17% of the variance in the pain intensity scores (R^2^ = 0.17, adjusted R^2^ = 0.14). Neuropathic pain was significantly and positively associated with pain intensity (*b* = 4.10, *p* < 0.001), indicating that individuals with neuropathic pain reported PROMIS pain intensity scores that were greater than four points higher than participants with non-neuropathic pain. Birth sex did not have a significant relationship; however, age showed a significant negative relationship with pain intensity [*b* = −0.08, 95% CI (1.99, 6.37), *p* = 0.04], with older participants experiencing slightly lower pain intensity. The overall pain interference model was statistically significant, F (3, 99) = 6.31, *p* < 0.001, and accounted for approximately 16% of the variance in the pain interference scores (R^2^ = 0.16, adjusted R^2^ = 0.14). Neuropathic pain was significantly associated with pain interference [*b* = 4.95, 95% CI (2.38, 7.36), *p* < 0.001], indicating that individuals with neuropathic pain reported PROMIS pain interference scores that were nearly five points higher than participants with non-neuropathic pain. Neither birth sex nor age had a significant relationship with pain interference.

## Discussion

This study highlights relationships between multiple types of cannabis use and experiences of neuropathic and non-neuropathic pain. In this sample of adults recruited from an urban pain specialty clinic, those individuals most bothered by neuropathic pain report using cannabis more frequently, both in terms of days per month and times per day. Notably, these adults were more likely to use THC-only and combination THC/CBD products. Because previous studies found THC products to be more effective in managing neuropathic pain by interacting with the endocannabinoid system (8, 9), it is possible that our participants also experienced benefit; this could explain their higher use of THC containing products.

Participants whose most bothersome pain was neuropathic pain showed a preference for combination THC/CBD products and used edibles, whole flower, and topicals more frequently than those with non-neuropathic pain. This preference may reflect the longer-lasting effects and ease of use associated with edible products. Concentrates, which contain higher levels of cannabinoids, were also more frequently used by participants whose most bothersome pain was neuropathic pain compared to non-neuropathic pain.

Adults whose most bothersome pain was neuropathic pain may use cannabis more frequently due to unique physiological processes that limit the effectiveness of conventional treatments (17). Although less evidence supports the benefits of cannabis in treating non-neuropathic pain, the interaction between cannabis and CB1 receptors in the nervous system may provide more targeted relief for neuropathic pain (26, 27). Such a mechanism could explain the reduced cannabis use among those with non-neuropathic pain. It is worth noting that both groups reported using THC/CBD combination products most commonly and on the most days of the month, with adults whose most bothersome pain was neuropathic pain averaging 26 days per month and non-neuropathic pain averaging 20 days per month. It is also notable that those with neuropathic pain in this sample reported higher levels of pain intensity and pain interference. Potentially, their higher symptom burden contributed to more cannabis use and could represent greater pain management needs.

Future research should examine whether the high frequency of cannabis use observed among adults whose most bothersome pain was neuropathic reflects gaps in the effectiveness of conventional treatments, and whether cannabis has merit as an adjunctive therapy. In our sample, these individuals reported using cannabis on an average of 26 days per month, compared to 20 days per month among those with non-neuropathic pain, suggesting that many are engaging in near-daily or daily use. This frequency raises important concerns, as daily or near-daily cannabis use has been associated with adverse outcomes, including increased risk for cannabis use disorder (CUD), dependency, and cardiovascular effects (28–30). Data from the Cannabis Use Disorders Identification Test-Revised (CUDIT-R) classify “4 or more times a week” as the highest risk category for use frequency, and epidemiological research suggests that roughly 1 in 10 regular users may develop CUD (29). Moreover, a recent study found that near-daily cannabis use significantly increases the risk of cardiovascular complications (30). Clinicians should be aware of these patterns and consider both potential therapeutic benefits and associated risks when discussing cannabis with patients. Tailoring product recommendations based on pharmacokinetics and patient-specific needs remains essential, but close monitoring is warranted, particularly for those with neuropathic pain who may be self-medicating with frequent use. These findings underscore the importance of integrating cannabis use screening and harm reduction strategies into routine pain management.

Within this sample, the findings suggest cannabis products rich in THC or with a balanced THC/CBD ratio are desirable within populations with neuropathic pain who are experiencing high levels of pain intensity and pain interference. It is possible that cannabis is providing some benefit or at least is perceived as beneficial. Individualized assessments are essential due to the uncertain long-term safety of frequent cannabis use, which raises concerns about cognitive impairment, tolerance, and dependency, necessitating patient education on responsible use, monitoring for overuse, and periodic breaks to prevent tolerance. These steps are important if cannabis is to be considered as a safe and effective adjunct to conventional pain therapies (31).

In addition to cannabis use patterns, this study also examined differences in pain symptom burden between individuals with neuropathic and non-neuropathic pain. Using validated PROMIS measures, we found that individuals who reported neuropathic pain as their most bothersome condition also reported significantly higher pain intensity and interference scores compared to those with non-neuropathic pain, even after controlling for age and sex. These findings align with prior literature suggesting that neuropathic pain is associated with higher symptom burden and greater functional impact (32, 33). The elevated pain burden among those with neuropathic pain may help explain their more frequent cannabis use, particularly of THC-rich products. It is possible that more nuanced pain assessments are needed that highlight the importance of considering both pain type and symptom severity when evaluating cannabis use behavior in chronic pain populations.

The study's cross-sectional design and reliance on self-reported data limit causal interpretations and may introduce recall or selection bias (34). Pain type classification was determined using a screening instrument rather than clinician verification, and cannabinoid potency, dose, formulation, and route were not standardized, which constrains interpretation at the product level. While the ID-Pain tool demonstrated strong diagnostic performance, it should complement, not replace, clinical judgment (35). This analysis reflects an interim cross-sectional evaluation of an ongoing longitudinal study; therefore, subgroup findings should be interpreted as exploratory and confirmed in larger samples. The greater frequency of cannabis use observed among individuals with neuropathic pain may reflect their higher symptom burden rather than a difference in efficacy or preference, and causal inferences cannot be made.

Yet, the confidential collection of survey data allowed for capturing rare information about cannabis use among adults with pain. Importantly, we highlight higher cannabis use frequency and pain symptom burdens among adults who identify neuropathic pain as their most bothersome pain. Clinicians may wish to heighten awareness of any unmet needs within this patient population and proactively ask about cannabis use. Further research with randomized, blinded designs is needed to clarify whether increased cannabis use in neuropathic pain leads to improved outcomes or adverse effects, as well as to optimize cannabis use in pain management by balancing its benefits and risks.

## Data Availability

The original contributions presented in the study are included in the article. Further inquiries may be directed to the corresponding author and may be made available upon reasonable request following approval of release policies and protocols.
